# Evaluation of Digital Breast Tomosynthesis as Replacement of Full-Field Digital Mammography Using an In Silico Imaging Trial

**DOI:** 10.1001/jamanetworkopen.2018.5474

**Published:** 2018-11-30

**Authors:** Aldo Badano, Christian G. Graff, Andreu Badal, Diksha Sharma, Rongping Zeng, Frank W. Samuelson, Stephen J. Glick, Kyle J. Myers

**Affiliations:** 1 Division of Imaging, Diagnostics, and Software Reliability, Office of Science and Engineering Laboratories, Center for Devices and Radiological Health, US Food and Drug Administration, Silver Spring, Maryland

## Abstract

**Importance:**

Expensive and lengthy clinical trials can delay regulatory evaluation of innovative technologies, affecting patient access to high-quality medical products. Simulation is increasingly being used in product development but rarely in regulatory applications.

**Objectives:**

To conduct a computer-simulated imaging trial evaluating digital breast tomosynthesis (DBT) as a replacement for digital mammography (DM) and to compare the results with a comparative clinical trial.

**Design, Setting, and Participants:**

The simulated Virtual Imaging Clinical Trial for Regulatory Evaluation (VICTRE) trial was designed to replicate a clinical trial that used human patients and radiologists. Images obtained with in silico versions of DM and DBT systems via fast Monte Carlo x-ray transport were interpreted by a computational reader detecting the presence of lesions. A total of 2986 synthetic image–based virtual patients with breast sizes and radiographic densities representative of a screening population and compressed thicknesses from 3.5 to 6 cm were generated using an analytic approach in which anatomical structures are randomly created within a predefined breast volume and compressed in the craniocaudal orientation. A positive cohort contained a digitally inserted microcalcification cluster or spiculated mass.

**Main Outcomes and Measures:**

The trial end point was the difference in area under the receiver operating characteristic curve between modalities for lesion detection. The trial was sized for an SE of 0.01 in the change in area under the curve (AUC), half the uncertainty in the comparative clinical trial.

**Results:**

In this trial, computational readers analyzed 31 055 DM and 27 960 DBT cases from 2986 virtual patients with the following Breast Imaging Reporting and Data System densities: 286 (9.6%) extremely dense, 1200 (40.2%) heterogeneously dense, 1200 (40.2%) scattered fibroglandular densities, and 300 (10.0%) almost entirely fat. The mean (SE) change in AUC was 0.0587 (0.0062) (*P* < .001) in favor of DBT. The change in AUC was larger for masses (mean [SE], 0.0903 [0.008]) than for calcifications (mean [SE], 0.0268 [0.004]), which was consistent with the findings of the comparative trial (mean [SE], 0.065 [0.017] for masses and −0.047 [0.032] for calcifications).

**Conclusions and Relevance:**

The results of the simulated VICTRE trial are consistent with the performance seen in the comparative trial. While further research is needed to assess the generalizability of these findings, in silico imaging trials represent a viable source of regulatory evidence for imaging devices.

## Key Points

**Question:**

Can in silico imaging trials play a role in the evaluation of new medical imaging systems?

**Findings:**

This diagnostic study used computer-simulated imaging of 2986 synthetic image–based virtual patients to compare digital mammography and digital breast tomosynthesis and found an improved lesion detection performance favoring tomosynthesis for all breast sizes and lesion types. The increased performance for tomosynthesis was consistent with results from a comparative trial using human patients and radiologists.

**Meaning:**

The study’s findings suggest that in silico imaging trials and imaging system computer simulation tools can in some cases be considered viable sources of evidence for the regulatory evaluation of imaging devices.

## Teaser

This diagnostic study uses computer-simulated imaging to evaluate digital breast tomosynthesis vs digital mammography for the detection of lesions.

## Introduction

Expensive and lengthy clinical trials for imaging products often hinder regulatory evaluation, are burdensome, and delay patient access to novel, high-quality devices. The evaluation of novel imaging technologies for screening typically requires a substantial clinical trial to demonstrate benefits compared with the standard of care.^1^ For example, regulatory approvals of digital breast tomosynthesis (DBT) are supported by clinical trials involving significant resources. A recent submission to the US Food and Drug Administration (FDA) of a DBT system as a replacement for digital mammography (DM), for example,^2^ relied on a clinical trial involving 400 women in 7 clinical sites receiving double exposure to ionizing radiation over many years, with 31 participating radiologists. We refer to this trial as the comparative trial. Leveraging recent progress in computer modeling and simulation as an alternative source of regulatory evidence with growing widespread support,^3,4,5^ we report on an in silico replication of the comparative trial to demonstrate the potential of this emerging approach.

While computational models are used to some extent in the regulatory evaluation of medical devices,^6^ their use in imaging has been rare. Models of imaging systems have significantly improved since the late 1980s,^7,8,9^ with sophisticated simulation tools increasingly used in research and development. In the last decade, powerful, efficient, and open-source radiation imaging system simulation tools have become freely available, allowing for a greater understanding of design choices. Recent efforts have established that computational methods can model many of the characteristics of breast anatomy and pathology^10,11^ as well as the physics of imaging.^12,13^ In addition, image interpretation algorithms have been shown to track the performance of human readers for specific visual tasks.^14^ While further research to advance in silico methods is needed, all elements required to perform an in silico imaging trial are rapidly approaching, or have already achieved, mature development stages warranting investigations into their use in replacing traditional clinical studies.

We report an all–in silico replication of a previously conducted imaging clinical trial used in support of the regulatory evaluation of DBT as a replacement for DM in breast cancer screening. Although studies have described models of DBT and DM systems,^15,16^ we know of no report of a computer-simulated imaging trial that replicates in size and nature a trial performed in support of a regulatory evaluation for which all codes and data sets are freely available in open-source format. We first describe the in silico replica (the Virtual Imaging Clinical Trial for Regulatory Evaluation, or VICTRE trial), including the trial population, the physics of the imaging systems, and the image reconstruction and interpretation algorithms. We then compare the results of VICTRE with those obtained in the human comparative trial and examine the limitations, benefits, and cost savings of the in silico approach.

## Methods

### Imaging Protocol

The study followed the Standards for Reporting of Diagnostic Accuracy (STARD) reporting guideline. Because the VICTRE trial was entirely simulated and made use of no human subject data, review by the FDA’s institutional review board was not applicable per the agency’s internal standard operating procedures.

**Figure 1.**
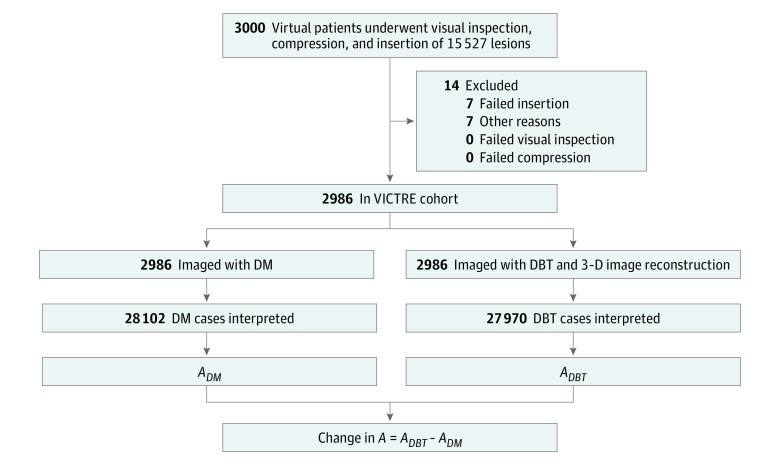
Virtual Patients’ Progress Through the Study Virtual patients underwent imaging with digital mammography (DM) and digital breast tomosynthesis (DBT). Multiple cases were obtained from each virtual patient image and used for image interpretation. *A* indicates the area under the receiver operating characteristic curve; 3-D, 3-dimensional.

Synthetic images of virtual patients were obtained using an in silico version of the Siemens Mammomat Inspiration^17^ DM and DBT system using a customized version of the MC-GPU Monte Carlo transport code^18^ (eAppendix 1 in the Supplement). The imaging parameters were selected based on publicly available device specifications and measurements^19^ for each compressed breast thickness. The Monte Carlo algorithm uses the known x-ray interaction physics and a stream of random numbers to sample a large number of x-ray tracks through the patient. Realistic models of the x-ray source and detector were created based on the technical specifications of the device being replicated. The simulation is physics based (no parameter was fitted to artificially force agreement on any performance metric). In the source model, the focal spot was modeled with a truncated Gaussian distribution with full width at half maximum equal to the nominal spot size of 0.3 mm. Radiographic spectra corresponded to 28 kilovolt (peak) (kV[p]) (for fatty and scattered breasts) and 30 kV(p) (for dense and heterogeneously dense breasts) from a tungsten anode with 50-μm rhodium and 1-mm beryllium filters.^20^ An analytical antiscatter grid^21^ was included in DM acquisitions (5:1 ratio, 31 line pairs/mm). The detector consisted of 2816 × 3584, 85-μm pixels with a 200-μm amorphous selenium transducer thickness. Depth of interaction and fluorescence emission in the selenium layer were explicitly calculated (eFigure 1 in the Supplement). Electronic noise was described using a Gaussian distribution with a variance of 5200 electrons.^22^ Pixel gain was set at 50 eV per detected charge with 0.99 Swank noise. The DBT system used 25 projections within a span of 50°. During DBT acquisition, x-ray tube motion was modeled by uniformly extending the focal spot along a 0.18° arc.^23^ Device simulations were accelerated using graphics processing unit computing^24^ and a delta-scattering transport algorithm. Variance reduction techniques were not used to preserve the realism of the quantum noise in the simulated images. Memory requirements were reduced using a binary tree voxel geometry.^24^ We reconstructed DBT volumes using a filtered back-projection algorithm.^25,26,27^ Because Siemens’ reconstruction algorithm is proprietary, we instead used a smoothing filter for a visually reasonable balance between sharpness and noise with negligible effect on reader performance.^16,28^ A flow diagram of the trial showing virtual patients’ progress through the study is presented in Figure 1.

### Trial Population

**Figure 2.**
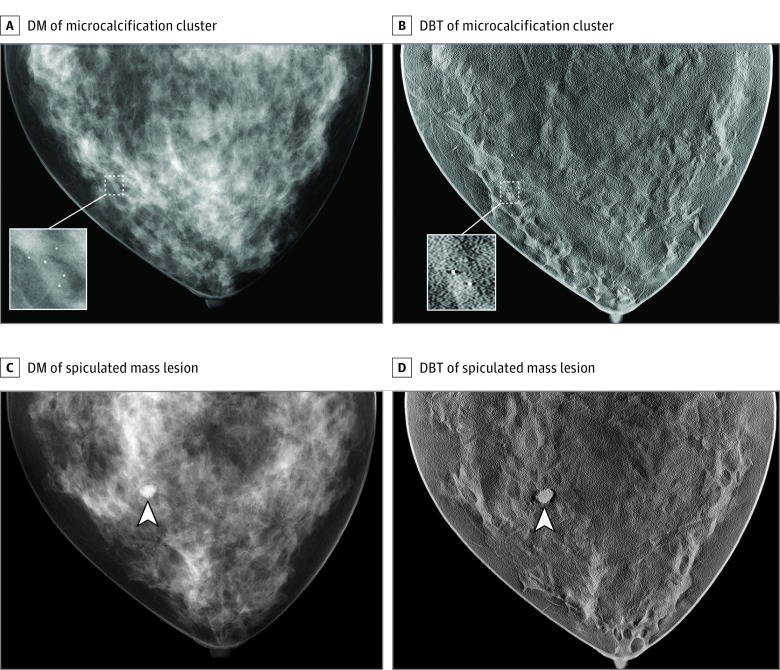
Example Images From the Virtual Imaging Clinical Trial for Regulatory Evaluation A and B, Digital mammography (DM) (A) and selected digital breast tomosynthesis (DBT) slice (B) of a case corresponding to a breast with scattered areas of fibroglandular density containing a microcalcification cluster (inserts). C and D, Digital mammography (C) and selected DBT slice (D) of a case corresponding to a breast with scattered areas of fibroglandular density containing a spiculated mass lesion (arrowheads). Lesions have been made more conspicuous for display purposes by artificially increasing their radiography attenuation during image acquisition.

The trial cohort consisted of virtual female patients whose breasts were generated using a procedural analytic model in which major anatomical structures (fat and glandular tissues, ductal tree, vasculature, and ligaments) are randomly generated within a predefined breast volume bounded by skin and chest wall^29^ at a voxel resolution of 50 μm (eAppendix 2, eFigure 2, and eFigure 3 in the Supplement). The model allows for control of patient characteristics such as breast volume, compressed thickness, and density, which are known to affect breast cancer lesion detection. Physical breast compression in craniocaudal orientation was performed using finite-element solid mechanics software.^30^ The breasts in this population fell into 4 density categories: extremely dense (0.548 glandular volume fraction [GVF]), heterogeneously dense (0.339 GVF), scattered fibroglandular densities (0.143 GVF), and almost entirely fat (0.071 GVF), with corresponding compressed thicknesses of 3.5, 4.5, 5.5, and 6 cm (Table 1). The positive cohort contained 2 types of lesions: a spiculated mass^11^ with a 5-mm nominal diameter and mass density 2% higher than normal glandular tissue and a microcalcification cluster consisting of 5 calcified lesions positioned within a 5-mm^3^ volume (eAppendix 2 in the Supplement). The calcifications were modeled as 195, 179, and 171 μm of solid calcium oxalate with a mass density scaled by 0.84 (1.78 g/cm^3^).^31^ The lesions were digitally inserted in a subset of the compressed breasts to create a positive cohort within each density category. To reduce computing time, up to 8 lesions (4 masses and 4 clustered microcalcifications) were inserted in approximately half of the virtual patients. The location of the inserted pathology was chosen randomly from candidate locations determined by the position of the terminal duct lobular units, a common site for carcinogenesis. Pathologies were nonoverlapping and did not extend into the chest wall or skin layer. Sample images of breast and lesions are presented in Figure 2. The lesion characteristics were adjusted during several prepilot stages to achieve a DM performance comparable to reported values.^1^

**Table 1.** Cohort Characteristics of the Virtual Imaging Clinical Trial for Regulatory Evaluation Population With Cases Corresponding to Regions of Interest in DM and Volumes of Interest in DBT by Breast Density Classes^a^

Virtual Patients and Cases	No. (%)				
	Total	Extremely Dense	Heterogeneously Dense	Scattered Fibroglandular Densities	Almost Entirely Fat
All virtual patients	2986 (100)	286 (9.6)	1200 (40.2)	1200 (40.2)	300 (10.0)
Virtual patients with lesion	1944 (100)	189 (9.7)	780 (40.1)	780 (40.1)	195 (10.0)
**DM Cases**					
Normal	15 527 (100)	1499 (9.7)	6237 (40.2)	6232 (40.1)	1559 (10.0)
With lesion	15 528 (100)	1499 (9.7)	6237 (40.2)	6232 (40.1)	1560 (10.0)
With spiculated mass	7756 (100)	747 (9.6)	3117 (40.2)	3112 (40.1)	780 (10.1)
With microcalcification cluster	7772 (100)	752 (9.8)	3120 (40.1)	3120 (40.1)	780 (10.0)
**DBT Cases**					
Normal	12 443 (100)	1244 (10)	4968 (39.9)	4996 (40.2)	1235 (9.9)
With lesion	15 527 (100)	1499 (9.7)	6237 (40.2)	6232 (40.1)	1559 (10.0)
With spiculated mass	7756 (100)	747 (9.6)	3117 (40.2)	3112 (40.1)	780 (10.0)
With microcalcification cluster	7772 (100)	752 (9.6)	3120 (40.1)	3120 (40.1)	780 (10.0)
Glandular volume fraction, mean (SD)^b^	0.256 (0.001)	0.548 (0.001)	0.339 (0.001)	0.143 (0.001)	0.071 (0.001)
Volume, mean (SD), cm^3^^b^	342.3 (0.001)	111.5 (0.001)	218.0 (0.001)	441.2 (0.001)	685.6 (0.001)
Compressed thickness, mean (SD), cm^b^	4.94 (0.001)	3.49 (0.001)	4.49 (0.001)	5.50 (0.001)	5.99 (0.001)

Abbreviations: DBT, digital breast tomosynthesis; DM, digital mammography.

^a^ According to Breast Imaging Reporting and Data System.

^b^ Based on a sample of 50 breasts per breast density class.

### Study Design

Approximately two-thirds of the trial virtual patients had a cancerous lesion corresponding to a microcalcification cluster or a spiculated mass. In silico cases corresponded to regions of interest extracted from DM images and DBT volumes. Analogously to the enrichment of cancer cases in the comparative trial population with patients found to have abnormal findings under a DM examination, the VICTRE trial population was designed to maximize the study’s statistical power. The target uncertainty was achieved by selecting an appropriate combination of number of diseased and normal cases, number of computational readers, and case difficulty. The design choices were to use the same number of readers as in the comparative trial (30) and vary the proportion of diseased cases to obtain sufficient cases for adequate number of training and testing sets.

Virtual patients were excluded if a computational error occurred during generation, compression, insertion of pathology, or acquisition and reconstruction of images. Exclusion criteria were enforced by monitoring software messages and by visually inspecting all images. Virtual patients whose compression failed to converge to target thicknesses (frequency of 0.03%) were replaced. In total, 14 of the 3000 virtual patients in the initial cohort were excluded.

The VICTRE trial was designed by the authors in consultation with a technical committee of industry, academia, and FDA representatives. The trial methods evolved in 2017 during several prepilot stages for model development, verification of input and output formats, and imaging parameters. All models used in the VICTRE pivotal trial were fixed prior to the pilot stage, which took place in the last half of 2017. The sponsor of the comparative trial had no role in the design and execution of the VICTRE trial.

### Image Interpretation

Images were interpreted by a computational reader under a location-known-exactly detection paradigm based on a channelized Hotelling observer^16,32,33^ using 5 Laguerre-Gauss channels with widths commensurate to lesion size.^34^ The Laguerre-Gauss channelized Hotelling observer is an efficient model observer and has been shown capable of trending human performance in detecting approximately round targets in backgrounds without strong directional texture.^28,35^ The computational reader for DBT used 3-dimensional channels by stacking the 2-dimensional channels for each slice following the volumetric approach of Platiša et al.^36^ For calcifications, spatial frequency filtering was used to adapt to irregular morphological features^37^ (eAppendix 3 and eFigure 4 in the Supplement). Thirty computational readers interpreted images (DM) and volumes (DBT) with (positive case) or without (negative case) a lesion. For each density group, 30 computational readers were trained with different sets of 100 pairs of positive and negative cases randomly sampled from a larger set of training pairs (260 for dense and fatty and 1000 for heterogeneously dense and scattered density breasts). For dense and fatty breasts, performance was tested on 360 negative and 500 positive cases. For heterogeneously dense and scattered density breasts, performance was tested on 1500 negative and 2000 positive cases. Using a fully crossed interpretation paradigm, all 30 virtual readers in the VICTRE trial interpreted all cases in the test data sets (eFigure 5 in the Supplement).

### Comparative Human Trial

The human trial used for the design of the VICTRE trial was conducted between 2012 and 2016, and submitted to the FDA in support of a premarket application for the approval of the DBT mode in Mammomat Inspiration^17^ as a replacement for DM (T. Mertelmeier, PhD, unpublished data, December 2016). Written informed consent was obtained from all participants in the study and institutional review board approvals were obtained from all collecting sites. The patient cohort in the comparative trial consisted of 326 asymptomatic women enriched based on DM, and double-exposed to radiation under both modalities. It included 21 patients with extremely dense breasts (5 positives), 156 with heterogeneously dense breasts (46 positives), 130 with scattered fibroglandular densities (41 positives), and 19 with almost entirely fatty breasts (10 positives). Follow-up examinations on 141 patients and biopsies on 83 patients were used to verify that the patients did not have malignant breast cancer, with biopsy-verified malignant cancer in 104 patients. The trial involved case selection from 7 clinical sites and took approximately 4 years to complete. Thirty-one certified radiologists reported 134 cancerous lesions, including 85 masses, 29 calcified lesions, 12 architectural distortions, and 8 asymmetric densities, with a total of 108 invasive cancers and 26 ductal carcinomas in situ. The trial reported 29 lesions of size less than 10 mm (21.6%), 40 lesions from 10 to 19 mm (29.9%), 33 lesions from 20 to 29 mm (24.6%), and 27 lesions larger than 30 mm (20.1%). The target, per-view, average (mean) glandular dose (AGD) for the comparative trial was approximately 1.0 and 1.5 mGy for DM and DBT, respectively. The comparative trial differential performance favored DBT by 0.043 (0.017), with area under the curve (AUC) measurements of 0.818 (0.019) and 0.861 (0.019) for 2-view DM and DBT, respectively (see Table 2 for subgroup outcomes).

**Table 2.** Trial End Points (AUC and Change in AUC) for DM and DBT per Lesion Type and per Radiographic Density With Radiation Dose Estimates for Each Subgroup and Breast-Level Nonparametric AUC for the Comparative Trial

Subgroup	AUC (SE)		Change in AUC (SE)^a^
	DM	DBT	
**VICTRE Trial**			
By size and radiographic density			
Total	0.9009 (0.0058)	0.9596 (0.0035)	0.0587 (0.0062)
Extremely dense	0.8358 (0.0127)	0.9020 (0.0106)	0.0657 (0.0148)
Heterogeneously dense	0.8643 (0.0067)	0.9372 (0.0042)	0.0724 (0.0073)
Scattered fibroglandular densities	0.9416 (0.0038)	0.9865 (0.0014)	0.0449 (0.0038)
Almost entirely fat	0.9475 (0.0061)	0.9975 (0.0014)	0.0500 (0.0061)
By lesion type			
Total for spiculated mass	0.8303 (0.0072)	0.9207 (0.0050)	0.0903 (0.008)
Extremely dense with spiculated mass	0.679 (0.018)	0.803 (0.015)	0.124 (0.021)
Heterogeneously dense with spiculated mass	0.760 (0.008)	0.876 (0.006)	0.116 (0.009)
Scattered fibroglandular with spiculated mass	0.902 (0.005)	0.975 (0.002)	0.073 (0.005)
Almost entirely fat with spiculated mass	0.972 (0.005)	0.996 (0.002)	0.024 (0.005)
Total for microcalcification cluster	0.971 (0.004)	0.9983 (0.0003)	0.0268 (0.004)
Extremely dense with microcalcification cluster	0.991 (0.002)	1.0000 (0.0001)	0.008 (0.002)
Heterogeneously dense with microcalcification cluster	0.968 (0.005)	0.9980 (0.0003)	0.029 (0.005)
Scattered fibroglandular with microcalcification cluster	0.981 (0.002)	0.9980 (0.0003)	0.017 (0.002)
Almost entirely fat with microcalcification cluster	0.923 (0.007)	0.9990 (0.0003)	0.076 (0.007)
**Comparative Trial**			
By size and radiographic density			
Total	0.818 (0.019)	0.861 (0.019)	0.043 (0.017)
Dense	0.802 (0.027)	0.844 (0.026)	0.043 (0.026)
Nondense	0.826 (0.026)	0.873 (0.026)	0.047 (0.021)
By lesion type			
Masses	0.858 (0.018)	0.923 (0.018)	0.065 (0.017)
Microcalcifications	0.796 (0.042)	0.749 (0.041)	−0.047 (0.032)

Abbreviations: AUC, area under the curve; DBT, digital breast tomosynthesis; DM, digital mammography; VICTRE, Virtual Imaging Clinical Trial for Regulatory Evaluation.

^a^ Change in AUC = AUC of DBT − AUC of DM.

### Trial End Points

The primary end point of the trial was the difference in AUC between DBT and DM corresponding to the entire patient population. In addition, we report subgroup analyses corresponding to the change in AUC for the 4 different breast classes and 2 lesion types.

### Statistical Analysis

The standard error of the change in AUC was estimated using a fully crossed (all virtual patients were imaged in both modalities), multiple-reader, multiple-case analysis using the iMRMC software (available at https://github.com/DIDSR/iMRMC).^38^ The trial was sized during a pilot study for an SE of 0.01 in change in AUC, lower than the uncertainty seen in the comparative human trial. To prevent bias, DBT performance was not analyzed as models were developed during prepilot stages. We considered 2-sided 95% confidence intervals (level of significance, *P* < .05).

## Results

In this simulated trial, computational readers analyzed 31 055 DM and 27 960 DBT cases from 2986 virtual patients with the following Breast Imaging Reporting and Data System densities: 286 (9.6%) extremely dense, 1200 (40.2%) heterogeneously dense, 1200 (40.2%) scattered fibroglandular densities, and 300 (10.0%) almost entirely fat.

A total of 2986 images of virtual patients were obtained for both modalities. The demographic characteristics of the cohort were designed to mirror the comparative trial in breast size, compressed thickness, and radiographic density (Table 1). The mean (SE) AGD for the trial population was 0.94 (0.04) mGy and 1.38 (0.06) mGy for DM and DBT, respectively. The AGD increased from dense (0.74 mGy for DM and 1.09 mGy for DBT) to fatty (1.14 mGy for DM and 1.66 mGy for DBT). Although relating in silico and comparative AGD estimates is not straightforward (eAppendix 4 in the Supplement), in silico AGD levels derived from automatic exposure control settings for virtual patients^39^ were within 15% of the AGD values in the comparative trial (eFigure 6 in the Supplement). Anatomical image textures were characterized by calculated power-law exponents^40^ (β [SE]) of 3.88 (0.20) and 2.45 (0.35) for DM and DBT, respectively, decreasing from dense to fatty breasts as expected. This reduction in β for DBT (approximately 0.7) is consistent with observations made on patient DM and DBT images^41^ (eAppendix 5 and eFigure 7 in the Supplement).

The VICTRE trial, including the generation of virtual patients, was performed between January and May 2018 and conducted in mixed-platform computer clusters containing a variety of central processing unit and graphics processing unit processors. On average, the simulation of each virtual patient took approximately 8 central processing unit–hours and 0.5 graphics processing unit–hours. The pivotal trial took approximately 2 weeks of computations. All code, parameters, and data sets are available at https://github.com/DIDSR/VICTRE (eAppendix 6 in the Supplement).

**Figure 3.**
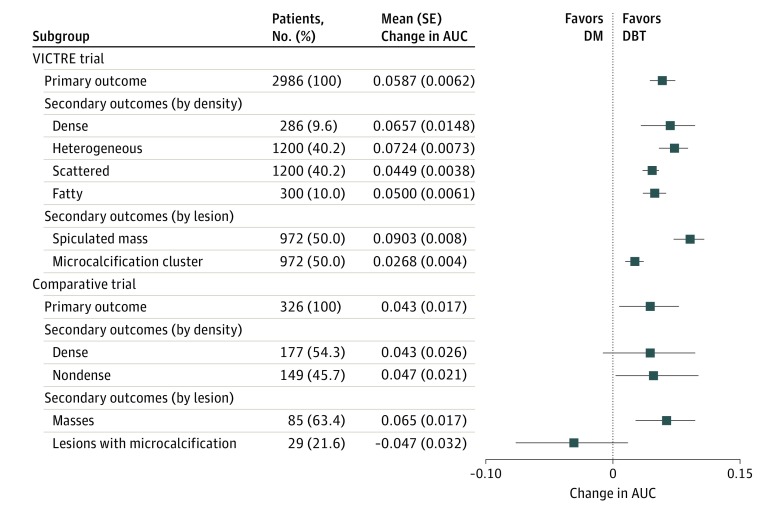
Trial Primary and Secondary Outcomes AUC indicates area under the receiver operating characteristic curve; DBT, digital breast tomosynthesis; DM, digital mammography; VICTRE, Virtual Imaging Clinical Trial for Regulatory Evaluation; and error bars, standard errors.

We observed a mean (SE) AUC of 0.9005 (0.0058) for DM and 0.9596 (0.0035) for DBT with a mean (SE) change in AUC of 0.0587 (0.0062), which was statistically significant (*P* < .001) in favor of DBT. The differential performance favored DBT in all subgroups. The mean (SE) change in AUC was larger for masses (0.0903 [0.008]) than for calcifications (0.0268 [0.004]), which was consistent with the findings of the comparative trial (mean [SE], 0.065 [0.017] for masses and −0.047 [0.032] for calcifications). In addition, the change in AUC was larger for masses than for calcifications for all breast sizes and subgroups (Table 2 and Figure 3). The differential performance observed is consistent not only with the aggregated results of the comparative trial, but also in terms of a larger differential performance for detecting masses vs microcalcifications.

## Discussion

On one hand, traditional trials use devices in actual clinical environments, incorporate the complexity of patient anatomy and disease variability, and use end points that are directly associated with clinical use. On the other hand, in silico trials can encompass larger virtual patient cohorts and control sources of variability to efficiently highlight technological differences without augmenting patient risk from radiation exposure. The VICTRE in silico trial found that DBT outperformed DM for all lesions and types of breasts included in the study with changes in AUCs between 0.027 and 0.090. The results of the VICTRE trial are consistent with the overall differential performance observed in the comparative trial.

An observation not clearly seen in the comparative trial data is that DBT undoubtedly outperforms DM in detecting microcalcifications. Several factors might contribute to this observation. First, in the comparative trial, calcified lesions were defined as any lesion with a calcified finding. Second, the computational models in the VICTRE trial do not include patient motion; thus, small microcalcifications might appear sharper than in the comparative trial images. In addition, future developments in in silico imaging trials might have the potential to elucidate device comparisons that are challenging or impractical to investigate with human participants (for instance, due to very low prevalence), thus generating useful evidence beyond traditional trials.^42^ An additional difference worth noting is that the comparative trial used 2-view DBT and DM per breast, whereas VICTRE results are based on 1-view imaging.

Validating predictions of computational models is always challenging. This work builds on research that has led to increased confidence in the in silico tools. More generally, the FDA has recently issued guidance on reporting criteria for including computational modeling in device submissions.^43^ However, VICTRE and other in silico clinical trials have considerable advantages over traditional trials, including larger statistical power, due in part to the availability of larger trial population samples, the ability to study rare cases challenging to document in patients, and the study of prototype devices not yet available.

An additional and notable advantage of in silico clinical trials is the substantial savings in resources. A precise estimate of savings will only emerge once additional in silico trials are reported. While resource savings strongly depend on the specifics of the trial (eg, device characteristics, disease prevalence, and availability of target population), estimates indicate that VICTRE required one-third of the resources required for the comparative trial. The VICTRE trial’s expenditure in scientist-hours was comparable to that in the comparative trial (approximately 3 full-time staff). The comparative trial took approximately 4 years to complete, while VICTRE took 1.75 years. Operational costs of the comparative trial, including additional patient recruitment needed to maintain statistical power given participant nonadherence and dropout, were equal to, if not larger than, the computational costs associated with the VICTRE trial. In addition, the estimate disregards other savings, including the risk from double-exposing hundreds of trial participants to ionizing radiation and cost associated with institutional review board approvals, clinical site fees, patient recruitment, and follow-up expenses. In addition to being conservative, our savings estimate is likely to increase over time as computing resources become increasingly inexpensive and more widely available.

The all–in silico method is not intended to replace, but rather complement and minimize, traditional clinical trials. Incrementally incorporating computational results as prior knowledge in Bayesian trial designs decreases sample size and trial length in the evaluation of medical implants.^44^ In some cases, patient and medical practitioner involvement will likely remain essential.

### Limitations

The simulated VICTRE trial considered only one realization of a spiculated mass and a calcification cluster, neglecting lesion variability and 15% of the lesions in the comparative trial (9% architectural distortions and 6% asymmetries). A 3-dimensional imaging system that outperforms DM in detecting masses and calcifications might also perform well in detecting architectural distortions and asymmetries. A recent retrospective study of cases recommended for biopsy reported that architectural distortion was more commonly detected in DBT than in DM.^45^ In addition, although VICTRE considered a range of breast thicknesses and radiographic densities, the patient variability of its trial population was in many ways different than the variability seen in clinical trial populations. These and other limitations of the VICTRE trial should be further investigated, including the lack of visual search in the image interpretation^46^ and a trial outcome defined only as detection of lesions and not as probability of malignancy.

## Conclusions

The findings of the simulated VICTRE trial suggest that the regulatory assessment of the specific DM and DBT imaging devices based on in silico data would have been similar to the actual regulatory decision made based on the comparative trial. It is useful to note that the findings of the VICTRE trial are not directly generalizable to other implementations of DM and DBT imaging systems and that additional studies will require appropriate model adaptations. The VICTRE trial, performed exclusively with open-source computational methods, suggests that increased use of computational modeling tools in the regulatory assessment of imaging systems could significantly decrease the burden of bringing new and improved imaging technologies to market. The work reported in this article provides evidence that state-of-the-art computational methods, coupled with predictive methods and laboratory testing, can lead to less burdensome regulatory evaluation approaches. Further investigations will help provide necessary validation of the described approach when applied to the evaluation of various other medical products.

## Notes

Supplement. **eAppendix 1.** VICTRE Trial Imaging Physics **eFigure 1.** Comparison of Simulated and Experimental Modulation Transfer Function (MTF) of the VICTRE Detector in Different Configurations **eAppendix 2.** Virtual Patients in the VICTRE Trial **eFigure 2.** Anatomical Components of Virtual Breast Model **eFigure 3.** Some Breast Model Elements **eAppendix 3.** Implementation of the VICTRE Trial Computer Reader Algorithms **eFigure 4.** Flowchart of the Image Interpretation Process by the Computer Readers in VICTRE **eFigure 5.** The Typical Reader Templates Used in VICTRE for Detecting Lesions in DM and DBT **eAppendix 4.** Radiation Dose Estimates in the VICTRE Trial **eFigure 6.** Radiation Dose Distributions in the VICTRE Trial Population **eAppendix 5.** Power-Law Beta Estimates of VICTRE Trial Images **eFigure 7.** A Scatterplot of β_recon Versus β_mammo for the Four Breast Glandular Densities **eAppendix 6.** The VICTRE Trial Imaging Pipeline
