# Supplementary material for: Evaluation of Digital Breast Tomosynthesis as Replacement of Full-Field Digital Mammography Using an In Silico Imaging Trial
Source: JAMA Netw Open. 2018 Nov 30;1(7):e185474. doi: 10.1001/jamanetworkopen.2018.5474 (PMC6324392; doi:10.1001/jamanetworkopen.2018.5474)
Supplement: Supplement. — eAppendix 1. VICTRE Trial Imaging Physics eFigure 1. Comparison of Simulated and Experimental Modulation Transfer Function (MTF) of the VICTRE Detector in Different Configurations eAppendix 2. Virtual Patients in the VICTRE Trial eFigure 2. Anatomical Components of Virtual Breast Model eFigure 3. Some Breast Model Elements eAppendix 3. Implementation of the VICTRE Trial Computer Reader Algorithms eFigure 4. Flowchart of the Image Interpretation Process by the Computer Readers in VICTRE eFigure 5. The Typical Reader Templates Used in VICTRE for Detecting Lesions in DM and DBT eAppendix 4. Radiation Dose Estimates in the VICTRE Trial eFigure 6. Radiation Dose Distributions in the VICTRE Trial Population eAppendix 5. Power-Law Beta Estimates of VICTRE Trial Images eFigure 7. A Scatterplot of β_recon Versus β_mammo for the Four Breast Glandular Densities eAppendix 6. The VICTRE Trial Imaging Pipeline [file jamanetwopen-1-e185474-s001.pdf]

Supplementary Online Content

Badano A, Graff CG, Badal A, et al. Evaluation of digital breast tomosynthesis as replacement of full-field digital mammography using an in silico imaging trial. *JAMA Netw Open*. 2018;1(7):e185474. doi:10.1001/jamanetworkopen.2018.5474

eAppendix 1. VICTRE Trial Imaging Physics

eFigure 1. Comparison of Simulated and Experimental Modulation Transfer Function (MTF) of the VICTRE Detector in Different Configurations

eAppendix 2. Virtual Patients in the VICTRE Trial

eFigure 2. Anatomical Components of Virtual Breast Model

eFigure 3. Some Breast Model Elements

eAppendix 3. Implementation of the VICTRE Trial Computer Reader Algorithms

eFigure 4. Flowchart of the Image Interpretation Process by the Computer Readers in VICTRE

eFigure 5. The Typical Reader Templates Used in VICTRE for Detecting Lesions in DM and DBT

eAppendix 4. Radiation Dose Estimates in the VICTRE Trial

eFigure 6. Radiation Dose Distributions in the VICTRE Trial Population

eAppendix 5. Power-Law Beta Estimates of VICTRE Trial Images

eFigure 7. A Scatterplot of β_recon Versus β_mammo for the Four Breast Glandular Densities

eAppendix 6. The VICTRE Trial Imaging Pipeline

This supplementary material has been provided by the authors to give readers additional information about their work.

© 2018 Badano A et al. *JAMA Network Open.*

eAppendix 1: VICTRE trial imaging physics

The images in the VICTRE trial were generated with an in-silico version of the Siemens Mammomat Inspiration system based on the MC-GPU Monte Carlo transport code. The simulation code was developed using publicly available information on the specifications and operation of the clinical device. Our guiding principle while developing the code was to model all device components that are relevant to the image formation process with as much realism as possible. Reasonable approximations were made to increase the computational efficiency and to model parts of the device for which limited public information was available. The following describes the main models implemented in the code:

1. **X-ray source**
    a. **Energy spectrum.** The initial energy of each x ray was randomly sampled from a histogram of a 28 or 30 kVp Tungsten anode spectrum after 50-micron Rhodium and 1-mm Beryllium filtration (spectra generated using [23]).
    b. **Focal spot**. The initial location of each x ray was randomly sampled using a 3D Gaussian distribution (i.e., uniform distribution on the surface of a sphere with a Gaussian distributed radius) centered at the nominal focal spot location 65 cm above the detector. The full width at half maximum of the Gaussian distribution was 300 microns, corresponding to the nominal focal spot size of the device. The Gaussian distribution was cropped at 2 standard deviations to prevent the generation of x rays far from the nominal location [see S Prasad, WR Hendee, and PL Carson, Med. Phys. 3, p. 217-223, 1978].
    c. **Motion blur** (from continuous tube motion during DBT acquisitions): the x-ray location after focal spot sampling was rotated about the DBT system rotation axis using a randomly sampled angle following a uniformly distribution between -0.09 and +0.09 degrees. The total 0.18-degree arc motion corresponds to a combined effect of exposure time and angular rotation speed.
2. **X-ray detector**
    a. **Antiscatter grid.** An analytical model of a 1D, focused grid was implemented to randomly sample if an x ray arriving at the detector was absorbed by the grid. The grid model was used for DM acquisitions only. For DBT acquisitions, the grid was not used, and software-based scatter correction was not performed. The composition of the grid materials and other grid physical parameters were approximated.
    b. **Direct-conversion detector layer.** A 200-micron-thick amorphous Selenium layer was defined as the active detector. X rays are tracked inside the detector layer until a Compton or photoelectric interaction takes place. The entire x-ray energy is locally deposited at the pixel covering the interaction location. This model allows x rays to cross the detector without interaction and reproduces the angle-dependent point spread function. If the energy deposited in the interaction is larger than the main Selenium K-edge energy (12.6 keV), a fluorescence characteristic x ray is emitted (59.6% probability). This secondary x ray is tracked until escaping or until re-absorption contributing to image blurring and correlating neighboring pixels.

To validate the accuracy of the models implemented in MC-GPU simulations, standard bench testing experiments used to characterize imaging devices were reproduced in silico. The simulated bench testing is useful to estimate the sensitivity of the results to the approximations of the models. The Modulation Transfer Function (MTF) of the simulated detector was measured following the methodology described in the international standard IEC 62220-1-2 ("Characteristics of digital x-ray imaging devices – Detectors used in mammography"). Following this method, simulations of x-ray projections of a steel edge were performed using with the same exposure parameters used in the DM simulations. The detector MTF in different orientations was

© 2018 Badano et al. *JAMA Network Open.*

estimated from the edge images using publicly available software [Y. Kao, M. Albert, A. Carton, H. Bosmans, and A. Maidment, Proc. SPIE Medical Imaging 2005: Physics of Medical Imaging 5745 (2005)]. A comparison of MTFs from simulated images and experimental measurements [A. Makeev, L. Ikejimba, and S. J. Glick, SPIE Medical Imaging. 2018] are presented in Figure 3. The results show that the simulated detector MTF has a comparable shape and is only approximately 5% sharper than the experimental results.

The small discrepancy in the MTFs is mostly caused by three approximations in the simulation code: ignoring electron transport and not modeling the secondary charge diffusion inside the Selenium layer, simple modeling of Selenium fluorescence using a single average characteristic ray energy with uncertainty in the fluorescence yield, and lack of additional scattering from detector components other than the sensitive layer which cannot be avoided in the experimental setting. The simulated MTFs at different acquisition angles and with motion blur agree well with experimental measurements reported in the literature. The MTF is typically defined only for normal acquisition with a static source. Analyzing images of the edge in other angular conditions is valuable to assess the model under tomosynthesis projections.

eFigure 1: Comparison of simulated and experimental modulation transfer function (MTF) of the VICTRE detector in different configurations. The vertical red line indicates the Nyquist frequency of the detector given by the pixel size of 85 microns.

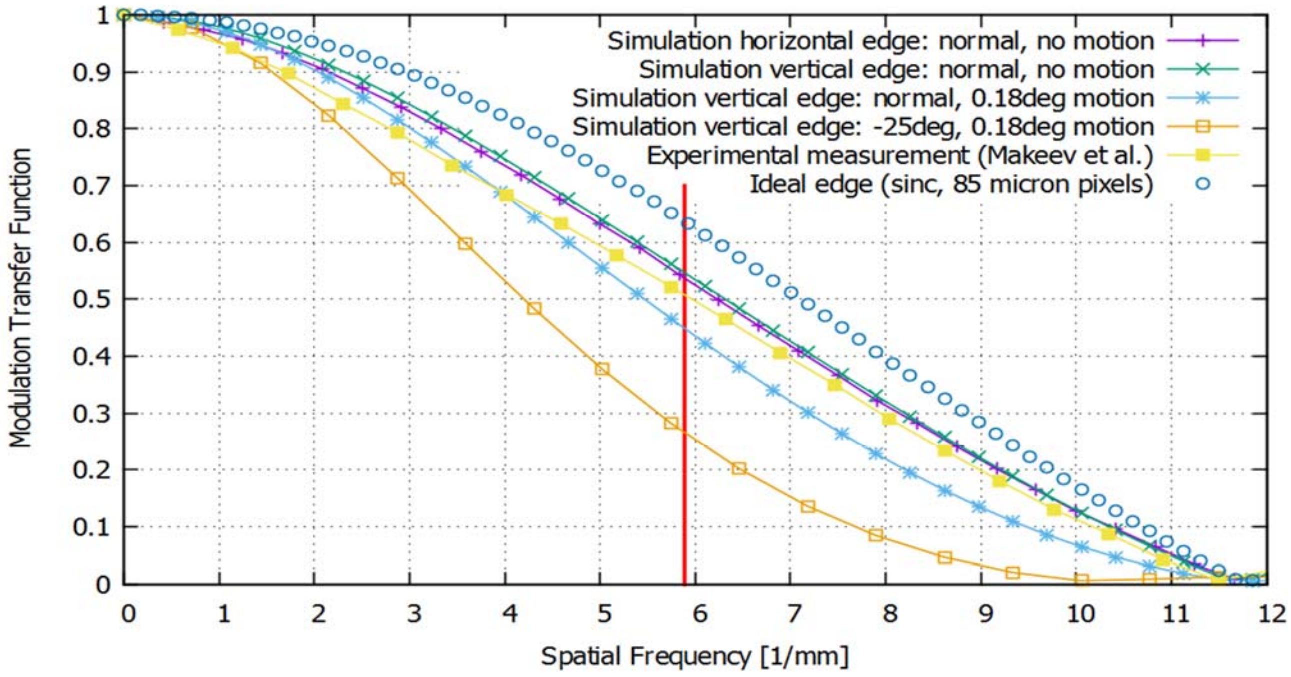

© 2018 Badano et al. *JAMA Network Open.*

eAppendix 2: Virtual patients in the VICTRE trial

The breast models in VICTRE are based on the method described in detail by Graff (C. G. Graff, *Proc. SPIE Medical Imaging*, San Diego, 2016). An overview of the model is illustrated in eFigure 2. The outside surface is first formed by a quadratic hemisphere shell with a skin layer and nipple area overlaid. The shape of the shell is adjusted for overall breast volume and surface curvature. Voronoi segmentation was used to randomly divide the interior of the shell into regions of fat or glandular components, with each glandular component containing a ductal network with terminal duct lobular units (see eFigure 3). The volume is then filled with Cooper's ligaments, chest muscle, and blood vessels. To physically compress the breast object, the volume is transformed into a tetrahedral mesh and each mesh element as given elastic properties determined by the glandular or adipose voxels at the center of the element. The mesh is then deformed using linear elasticity finite element modeling with the breast compressed in a craneocaudal orientation to a thickness of choice. For the VICTRE trial, the breast model was sampled at 50 µm, isotropic voxel size. The implementation is initiated with a set of random numbers and creates random voxelized breast anatomy objects segmented into 9 different tissue types. Several different modeling techniques are employed including a non-isotropic Voronoi segmentation, recursive tree branching algorithms to generate a ductal tree and vascular network, and Perlin-noise perturbed random spheroids to create fat lobules.

eFigure 2: Anatomical components of virtual breast model.

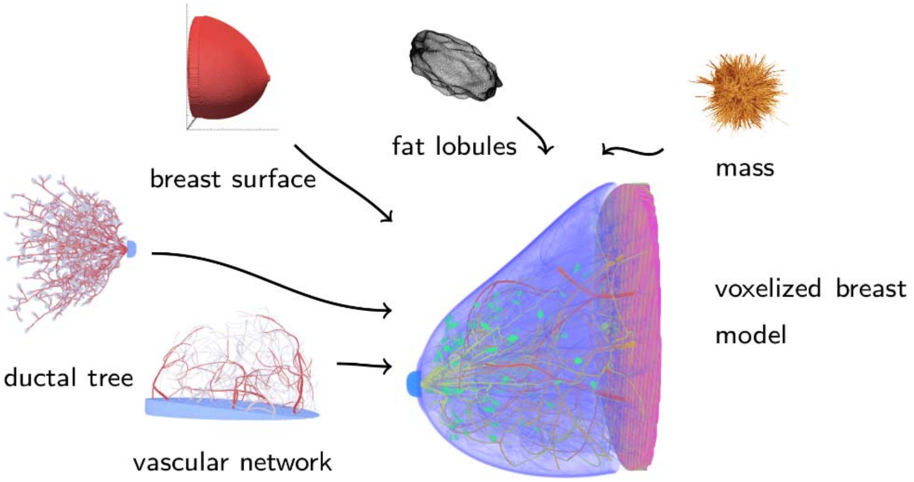

eFigure 3: Some breast model elements: (a) vascular network, and (b) ductal tree with terminating lobular units.

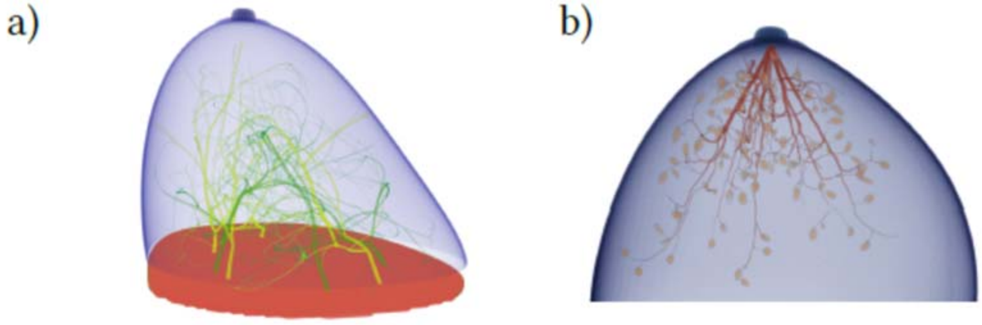

© 2018 Badano et al. *JAMA Network Open.*

eAppendix 3: Implementation of the VICTRE trial computer reader algorithms

The reader model was designed based on a channelized Hotelling model observer (CHO) for a location-known detection task. The CHO is a type of linear model observer (MO) which calculates a probability score (t) of an image (g) by multiplying a template w with the image ($t = w'g$). Depending on the mechanism of the MO, the template considers the statistics of the image differently. For the Hotelling observer (the optimal linear MO), the mean of signal (S) to be detected and the data covariance (K_g) are used to construct the MO's template as $w = K_g{}^{-1}S$. The mean and covariance are usually learned from a set of training cases. The process of forming the MO template is called training of the MO. After training, the MO is ready to be applied to a set of testing cases to evaluate the detection performance. eFigure 4 illustrates the reading process used in VICTRE including the training and testing of a MO and the output of an AUC (or detectability SNR) estimated from the distributions of the scores for a set of lesion-present and lesion-absent testing cases. For the CHO, channelization functions are applied to an image first to extract certain features used to derive the image score.

An advantage of channelization is the reduction of data dimensionality leading to significantly reduced data demands for MO training. Our reader model used five Laguerre-Gauss (LG) channels. LG-CHO has been shown to trend human performance for detecting a spherical lesion in backgrounds with no orientation. Because only five channels were used, 100 pairs of cases for training were sufficient. The reader model also adjusted to the signal type and imaging modalities. For DM, a 2D CHO was used. For DBT, a 3D CHO was used since readers acted on volume data. The LG channels for the spiculated mass had a Gaussian width of 30 and 25 pixels for DM and DBT respectively. Our 5 convolutional LG channels for clusters had a Gaussian width of 1.5 pixels. eFigure 5 shows the typical MO templates used in VICTRE for detecting the two types of lesions in DM and DBT. Note that the MO templates had the same dimensionality as the ROIs of images (DM) or volumes (DBT) extracted for evaluating lesion detection performance.

eFigure 4: Flowchart of the image interpretation process by the computer readers in VICTRE.

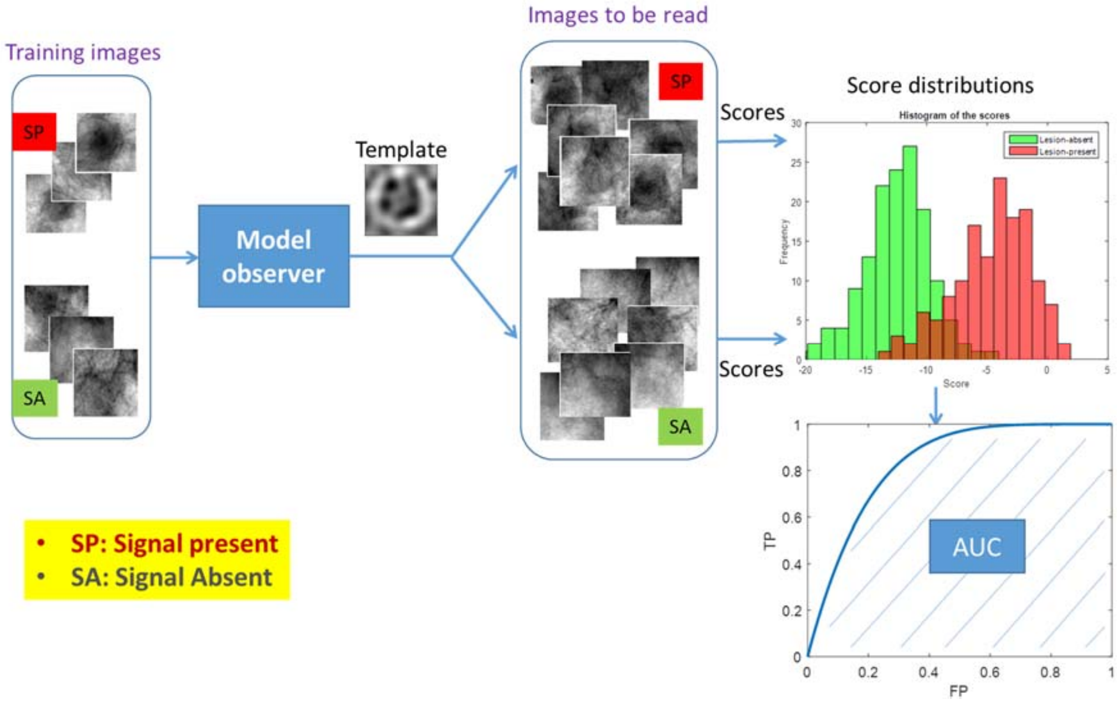

eFigure 5: The typical reader templates used in VICTRE for detecting lesions in DM and DBT. The middle column shows mean signals obtained by averaging 100 pairs of training patches. The right column shows the corresponding reader templates. The sizes for the patches are 109x109, 65x65, 109x109x9, and 65x65x5 from the top to the bottom row.

Lesion/Modality	Mean Signal	Reader's template
Spiculated mass in DM	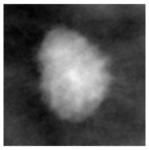	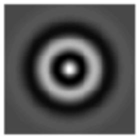
Spiculated mass in DBT	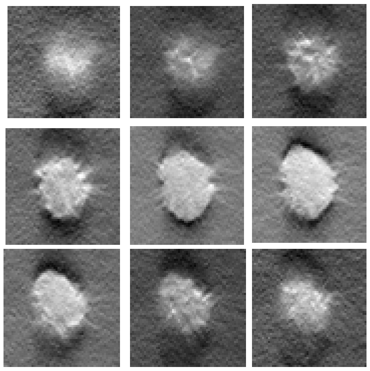	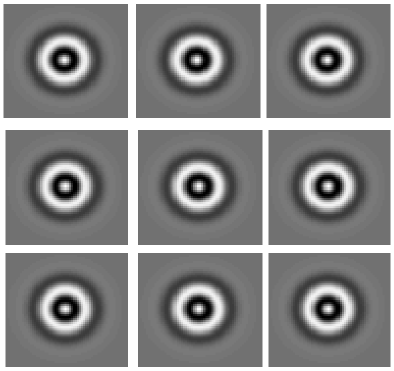
Clustered microcalcifications in DM	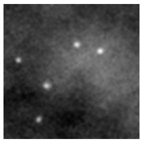	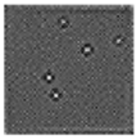
Clustered microcalcifications in DBT	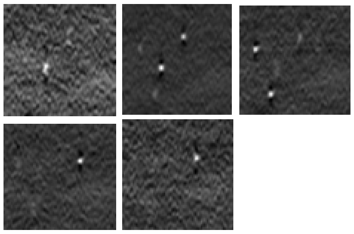	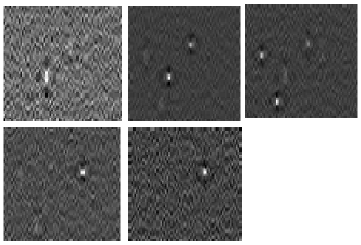

© 2018 Badano et al. *JAMA Network Open.*

eAppendix 4: Radiation dose estimates in the VICTRE trial

The distribution of radiation dose to patients in the comparative trial is only known as an average target. For the VICTRE trial we have exact estimates based directly on the same Monte Carlo simulations performed for the image acquisition in DM and DBT. The distribution of mean average glandular dose for the entire VICTRE population is presented in eFigure 6. Note that the match with the target, per-view, average glandular dose (AGD) for the comparative trial population (1.0 and 1.5 mGy for DM and DBT respectively) is reasonable (within 15%).

eFigure 6: Radiation dose distributions in the VICTRE trial population. Glandular dose for all virtual patients was calculated and included in this histogram for digital mammography (DM) and digital breast tomosynthesis (DBT) and for each of the four breast sizes and radiographic densities considered.

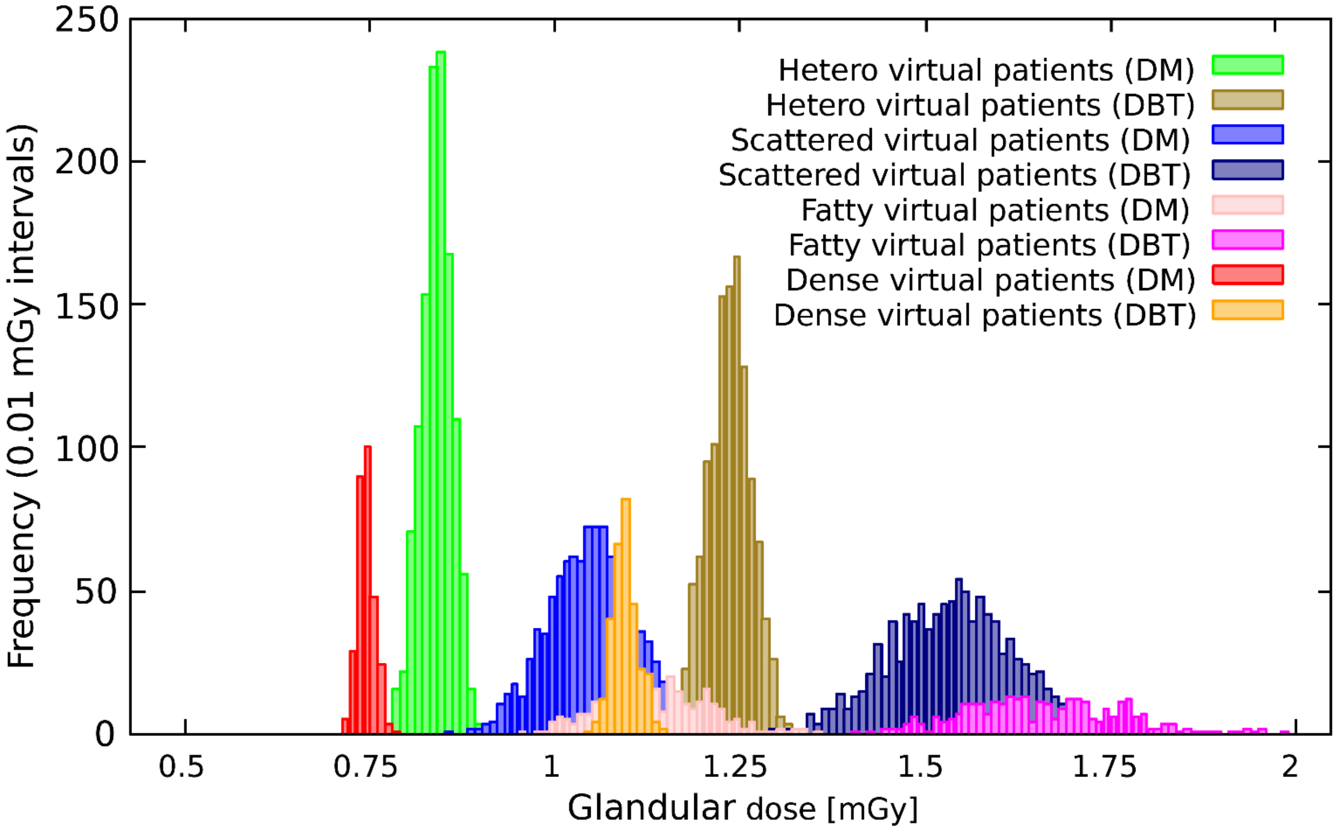

© 2018 Badano et al. *JAMA Network Open.*

eAppendix 5: Power-law beta estimates of VICTRE trial images

To assess the realism of the simulated images used in this study a power spectrum analysis was performed. It is well known that mammographic images have an exponentially decaying power spectrum P over a range of spatial frequencies f that represent anatomical structures (A. E. Burgess et al., “Human observer detection experiments with mammograms and power-law noise”, Med. Phys. (28)4, 2001), i.e.,

$$P(f) = \alpha e^{-\beta f}$$

The exponential decay constant β is commonly used to characterize and compare breast image texture. Following the procedure described by Burgess (A. E. Burgess, Proc. SPIE Medical Imaging Symposium, San Diego, 1999), β values were calculated for both mammographic and DBT reconstructed images across each of the four breast density categories. These values were compared to measurements reported for clinical datasets acquired using the same imaging system (L. Cockmartin et al., Med. Phys. 40(8), 2013). eFigure 7 summarizes this analysis. In general, there was good agreement between β for real and simulated images. The mean β for simulated mammograms was 3.88 versus 3.37 for the clinical population. For DBT reconstructed images mean β was 2.45 for simulated and 2.31 for real images. The distribution of β for the virtual patient DBT images was very consistent with the clinical distribution (eFigure 7c) while β for the virtual patient mammograms was slightly higher on average (eFigure 7b), indicating relatively lower high frequency mammographic content. This may be due to differences in the virtual patient population and the population studied by Cockmartin et al., as the virtual patient population was not designed to match the patient population in their study. The virtual population for example did not contain examples of extremely thick breasts or moderately thick dense breasts, which have more overlapping glandular tissue and ligaments, potentially creating more high frequency content in mammograms that would reduce average β. In principle prior knowledge of the β distribution for a patient population could be used to customize a virtual patient population, though that was not attempted in this work. General trends observed in clinical images were also present in the simulated data, including lower β for DBT images compared to mammographic images and higher β for denser breast types.

eFigure 7: A scatterplot of β_recon versus β_mammo for the four breast glandular densities, fatty (yellow), scattered density (blue), heterogeneously dense (green), and very dense (red) is given in (a). Plots (b) and (c) compare distributions of β for the VICTRE virtual patient to patient data for mammography and DBT reconstructions respectively (solid bars represent ±SD and dashed bars indicate observed extrema).

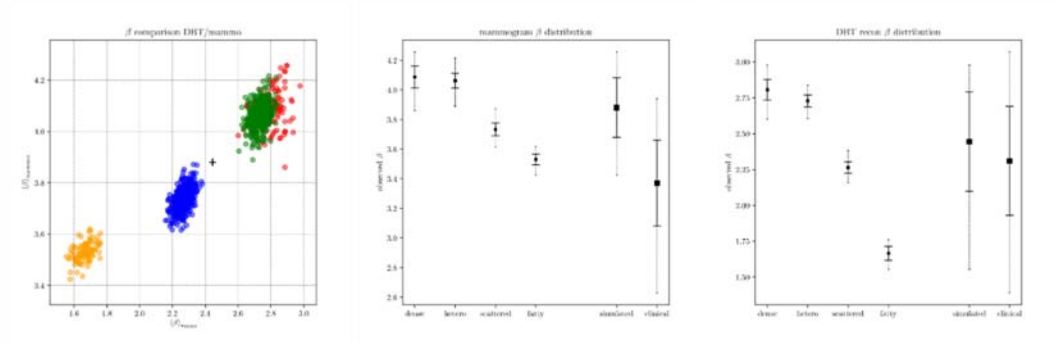

(a) (b) (c)

© 2018 Badano et al. *JAMA Network Open.*

eAppendix 6: The VICTRE trial imaging pipeline

All code, parameters, and datasets are available at https://github.com/DIDSR/VICTRE. Here we summarized VICTRE's pipeline, a complete imaging clinical trial software package. First, breast digital models are developed based on procedural approaches of normal anatomy. The breasts were imaged using GPU-accelerated Monte Carlo transport codes and interpreted using reader models for the presence of lesions. All these components were assembled into a cohesive computational pipeline and datasets made available in DICOM format for ease of visualization. We made available the pipeline and tools as an open-source collection in a VICTRE container. The container can be used either on local or cloud-based servers as-is or modified to execute a variety of imaging clinical trials.

Breast models (in-silico or virtual patients) are generated and cropped to a fixed volume to speed up loading and fit them in the limited Graphics Processing Units (GPU) memory. The breast generation and compression codes require input configuration files defining the parameters characterizing the breast shape, size, density, glandular fraction, and compression thickness. In addition to the model, the breast model code generates a file with lesion insertion location candidates based on the position of the terminal duct lobular units which are a common site for carcinogenesis. The digital breast phantom is saved in an 8-bit integer raw format file. The breast generation and compression codes are written in C++. Lesions are then inserted in a subset of the compressed breast phantom population to create cancer cases.

The lesion insertion locations are randomly chosen from the list of possible locations given by the breast phantom generation code. The selected location is then passed through checks to ensure that the lesion is within the phantom boundaries, non-overlapping with tissues like air, muscle, nipple, and skin, and nonoverlapping with already inserted lesions. The lesion insertion code uses the possible locations file generated by the breast model generation code as an input. Phantoms with lesions are then saved in the same raw format and lesion locations are saved in a text file. Lesion insertion code is developed using Python.

The in-silico patients are then imaged using a state-of-the-art Monte Carlo x-ray transport code (MC- GPU). We obtained projection images for the two modalities in the VICTRE trial: digital mammography (DM) and digital breast tomosynthesis (DBT). MC-GPU uses its own input configuration file describing imaging parameters including source, detector geometry, focal spot blur, and detector noise parameters. The output files are the DM images and DBT angular projections in 32-bit real little-endian raw format. The imaging code is written in C and CUDA (Compute Unified Device Architecture).

VICTRE implemented a filtered back-projection (FBP) reconstruction algorithm for DBT using single-threaded C modified from an extension of a single-threaded C code to allow for DBT reconstruction. The modifications account for an x-ray source moving in an arc about the object with a stationary detector with the z-axis of the object normal to the detector plane. The FBP reconstruction code input parameters including DBT volume details, distance from the source to detector, phantom dimensions, pixel pitch, and voxel size are provided via command line. The reconstructed volume is saved in 64-bit little-endian raw format.

After images are acquired, lesion-present and lesion-absent regions of interest (ROIs) are extracted from the DM images (or volumes of interest, VOIs, from the DBT volumes). Regions of interest are then interpreted by in-silico readers using a location-known exactly paradigm. For extracting lesion-absent ROIs, we applied rigorous checks including if the subimages are within the reconstructed volume boundaries and non-overlapping, to find appropriate locations. The ROI extraction code accepts input parameters via command line including phantom and DM and/or DBT image/volume details, and size and number of ROIs to be extracted. The output for this

© 2018 Badano et al. *JAMA Network Open.*

code (developed in Python) are extracted ROIs (or VOIs for DBT) in 32-bit real little-endian raw format, as well as their locations saved as a separate text file. These ROIs can then be read by a human or an in-silico reader for image interpretation.

Images from the VICTRE trial were generated in raw format and then converted to DICOM format. To accommodate the in-silico details, we added metadata in DICOM tags to describe the way the images were generated and to allow for reproducibility. We used tags including patient information, clinical trial description, imaging study performed per modality and series under each study. Breast type, lesion absence or presence, and compressed breast thickness are described as attributes of the patient undergoing the imaging studies. A complete description of the mapping of DICOM tags to the in-silico trial parameters, along with the dataset of all images generated in the VICTRE trial are publicly available via the Cancer Imaging Archive (https://wiki.cancerimagingarchive.net/display/Public/VICTRE).

© 2018 Badano et al. *JAMA Network Open.*
